# Correction: Development and evidence of validity of the HIV risk perception scale for young adults in a Hispanic-American context

**DOI:** 10.1371/journal.pone.0235212

**Published:** 2020-06-18

**Authors:** Patricio Mena-Chamorro, Rodrigo Ferrer-Urbina, Geraldy Sepúlveda-Páez, Francisca Cortés-Mercado, Carolina Gutierrez-Mamani, Kyara Lagos-Maldonado, María Peña-Daldo

Fig 2 is incorrect. The covariations of the dependent variables were omitted. The authors have provided a corrected version here.

**Fig 2:**
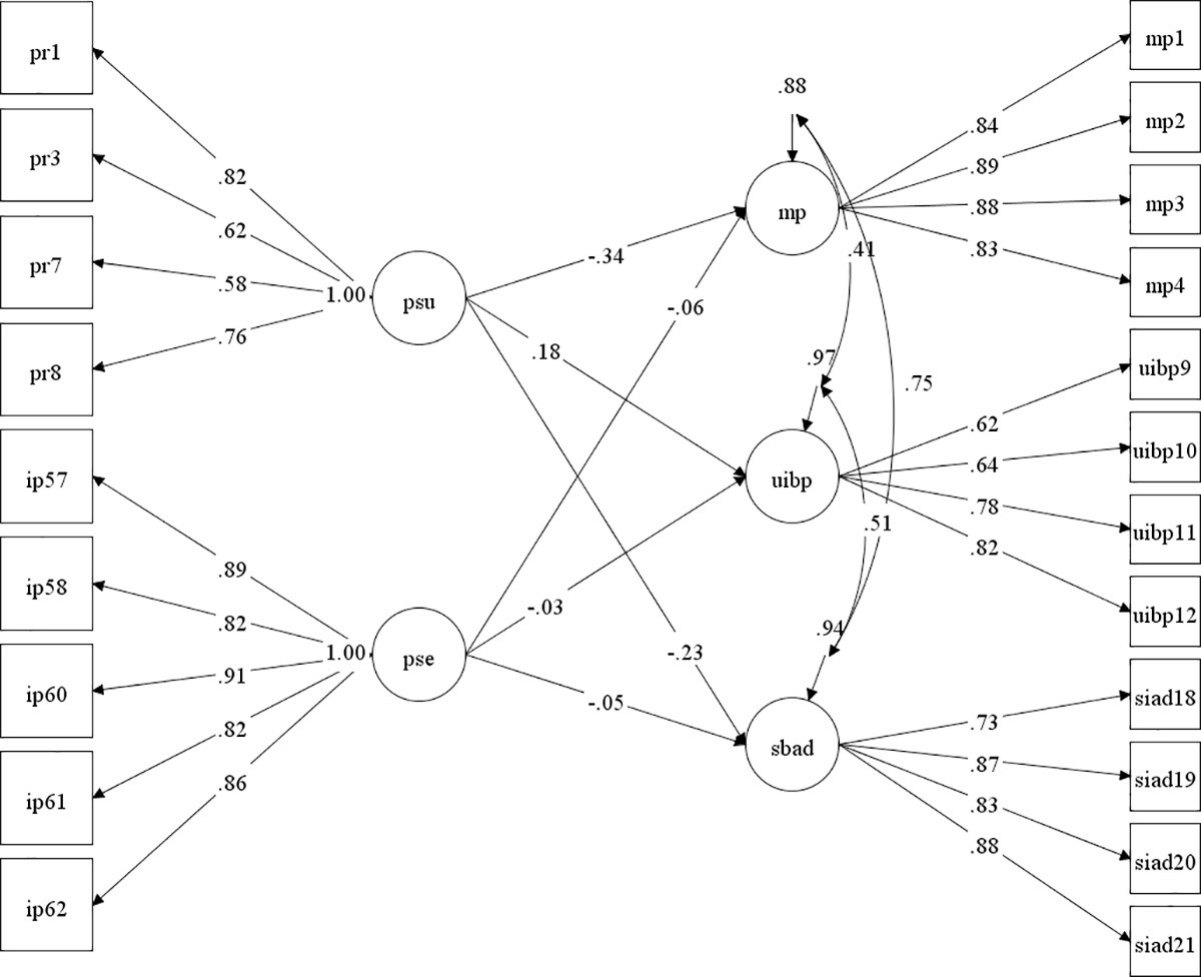
Graphical representation of the SEM model.
