# Supplementary material for: Development and evidence of validity of the HIV risk perception scale for young adults in a Hispanic-American context
Source: PLoS One. 2020 Apr 21;15(4):e0231558. doi: 10.1371/journal.pone.0231558 (PMC7173927; doi:10.1371/journal.pone.0231558)
Supplement: S1 Protocol — (PDF) [file pone.0231558.s001.pdf]

S1 Protocol: Spanish version scale

Estimada(o):

A continuación, se presentan una serie de declaraciones relacionadas con el VIH/SIDA. Por favor, indique en qué medida considera que estas afirmaciones son ciertas con respecto a su propia persona, es decir, si considera que es algo que le podría suceder a usted. Indique con un "X", el grado de acuerdo que usted tiene con las siguientes afirmaciones:

N.º	ÍTEMS	Falso	Parcialmente falso	Parcialmente verdadero	Verdadero
1	Podría contraer VIH/SIDA como cualquier otra persona.				
2	Podría ser portador de VIH sin saberlo.				
3	Podría estar contagiado de VIH y no presentar síntomas.				
4	Me preocupa infectarme de VIH/SIDA.				

A continuación, le pedimos que se imagine ser diagnosticado con el VIH / SIDA. Por favor, indique el grado en que cree que los siguientes ámbitos de su vida se verían afectados. Indique con un "X", el grado de acuerdo que usted tiene con las siguientes afirmaciones de acuerdo a:

En nada, si considera que no habría consecuencias negativas en ese ámbito.
Levemente, si considera que existiría algún perjuicio, pero que no afectaría en demasía su vida.
Moderadamente, si considera que existiría algún perjuicio importante, pero que podría sobrellevar.
Gravemente, sí cree que se perjudicaría demasiado y le sería muy difícil de sobrellevar.

N.º	ÍTEMS	En nada	Levemente	Moderadamente	Gravemente
1	Mi desarrollo personal.				
2	Mi vida laboral.				
3	Mi vida diaria.				
4	La relación con mis cercanos.				
5	Mis expectativas y metas a largo plazo.				
