# Supplementary material for: Development and evidence of validity of the HIV risk perception scale for young adults in a Hispanic-American context
Source: PLoS One. 2020 Apr 21;15(4):e0231558. doi: 10.1371/journal.pone.0231558 (PMC7173927; doi:10.1371/journal.pone.0231558)
Supplement: S2 Protocol — (PDF) [file pone.0231558.s002.pdf]

S2 Protocol: unofficial English translated scale (translation for guidance only)

Dear:

Below are a number of statements related to HIV/AIDS. Please indicate the extent to which you believe these statements to be true about your own person, that is, whether you believe this is something that could happen to you. Please mark with an "X" the degree to which you agree with the following statements:

N.º	ÍTEMS	False	Partially false	Partially true	True
1	You could get HIV/AIDS just like anyone else				
2	Could be HIV-positive without knowing it				
3	You could be infected with HIV and have no symptoms				
4	I'm worried about getting HIV/AIDS				

Next, we ask you to imagine being diagnosed with HIV/AIDS. Please state the degree to which you believe the following areas of your life would be affected. Please mark with an "X" the degree to which you agree with the following statements:

Not at all, if you consider that there would be no negative consequences in that area.
Slightly, if you consider that there would be some injury, but that it would not affect your life too much.
Moderately, if you consider that there would be some significant injury, but which you could overcome.
Seriously, if you believe that there would be too much harm and would be too difficult to bear.

N.º	ÍTEMS	Not at all	Slightly	Moderately	Seriously
1	My personal development				
2	My working life				
3	My daily life				
4	The relationship with my close ones				
5	My expectations and long-term goals				
