# Supplementary material for: Development and evidence of validity of the HIV risk perception scale for young adults in a Hispanic-American context
Source: PLoS One. 2020 Apr 21;15(4):e0231558. doi: 10.1371/journal.pone.0231558 (PMC7173927; doi:10.1371/journal.pone.0231558)
Supplement: S1 Table — (PDF) [file pone.0231558.s003.pdf]

S3 Table: Item description, standardized factor loadings, standardized factor cross-loadings and factorial covariations for each dimension in 23 items version

Original item<br>(untested translation for understanding purposes only)	PSu	PSe
Perceived Susceptibility of HIV		
Podría contraer VIH/SIDA como cualquier otra persona.<br>(You could get HIV/AIDS just like anyone else)	**.703****	.096
Relacionarme sexualmente con gente desconocida me vuelve más susceptible y propenso a contagiarme de ITS y/o VIH/SIDA.<br>(Having sexual relationships with strangers makes me more susceptible to getting STIs and/or HIV/AIDS.)	**.614****	**.164****
Podría ser portador de VIH sin saberlo.<br>(Could be HIV-positive without knowing it)	**.586****	-.014
Podría contagiarme de VIH/SIDA usando preservativo.<br>(I could get HIV/AIDS by using a condom.)	**.215****	-.024
Si mi pareja se cuida para evitar contagiarse de ITS y/o VIH/SIDA no es necesario que yo lo haga.<br>(If my partner takes care of himself to avoid getting STIs and/or HIV/AIDS, it is not necessary for me to do so.)	**.359****	.094
Mi pareja podría ser portadora de una ITS y/o VIH/SIDA sin necesariamente yo contagiarme.<br>(My partner could be a carrier of an STI and/or HIV/AIDS without me necessarily being infected.)	**.110****	**-.155****
Podría estar contagiado de SIDA y no presentar síntomas.<br>(You could be infected with AIDS and have no symptoms)	**.549****	.034
Me preocupa infectarme de VIH/SIDA.<br>(I'm worried about getting HIV/AIDS)	**.612****	**.158****
Perceived Severity of HIV		
Mi calidad de vida.<br>(My quality of life.)	**.306****	**.671****
Mi vida sexual.<br>(My sex life.)	**.620****	**.648****
Mis encuentros sexuales.<br>(My sexual encounters.)	**.606****	**.587****
Mi vida familiar.<br>(My family life.)	.002	**.746****
Sería discriminado.<br>(I would be discriminated against.)	**.207****	**.603****
Estaría más propenso a morir por una enfermedad.<br>(He'd be more likely to die from a disease.)	**.299****	**.509****
Mi imagen social y la de mi/s pareja/s.<br>(My social image and that of my partner(s))	**.196****	**.681****
Mi situación económica y/o la de mi familia.<br>(My financial situation and/or that of my family.)	**-.207****	**.616****
Mis ganas de vivir.<br>(My desire to live.)	**-.198****	**.851****
Mi desarrollo personal.<br>(My personal development.)	**-.303****	**.922****
Mi vida laboral.<br>(My working life)	**-.330****	**.817****
Mi estado emocional.<br>(My emotional state.)	-.051	**.855***
Mi vida diaria.<br>(My daily life)	**-.214****	**.922***
La relación con mis cercanos.<br>(The relationship with my close ones)	**-.215****	**.836****
Mis expectativas y metas a largo plazo.<br>(My expectations and long-term goals)	**-.103****	**.887****
Factorial covariations	**.207****	
