# Supplementary material for: Development and evidence of validity of the HIV risk perception scale for young adults in a Hispanic-American context
Source: PLoS One. 2020 Apr 21;15(4):e0231558. doi: 10.1371/journal.pone.0231558 (PMC7173927; doi:10.1371/journal.pone.0231558)
Supplement: Attachment — Submitted filename: respuestaeditor291119.docx [file pone.0231558.s005.docx]

Dear Editor:

First of all, let me thank you for your consideration and offer my apologies for the omissions presented. I then proceed to respond to them point by point:

1- PLOS ONE has specific requirements for studies that are presenting a new method or tool as the primary focus, including a newly developed or modified questionnaire or scale (https://journals.plos.org/plosone/s/submission-guidelines#loc-methods-software-databases-and-tools.) One requirement is that the questionnaire or scale must be openly available under a license no more restrictive than CC BY. In light of this, before we proceed, please include a copy of your questionnaire or scale as a Supporting Information file or provide a link if it is available through an online repository.

RESPONSE: We have attached the scale developed in its original Spanish version (including an English translation), which will be freely available to readers and can be modified and adapted freely.

2- a) the recruitment date range (month and year).

RESPONSE: was added in the materials and methods section (procedure).

Line 174-176, “Eight fifth-year psychology students were trained to give instructions, answer participants' questions and apply the questionnaires in pencil and paper format, in the city of Arica between March and May 2018”

3- b) a description of any inclusion/exclusion criteria that were applied to participant recruitment.

RESPONSE: was added in the materials and methods section (Design and participants).

Line 131-133, “Inclusion criteria were 18 years of age or older (i.e. age of legal autonomy in Chile) and being an active student of higher education. No subsequent exclusion criteria were applied.”

4- c) a table of relevant demographic details

RESPONSE: A table with relevant demographic data has been incorporated in the materials and methods section (Design and participants).

5- d) a statement as to whether your sample can be considered representative of a larger population.

RESPONSE: a more detailed statement was added to discussion section.

Line 280-282, “The main constraint of this study corresponds to the use of a non-probabilistic sampling strategy (i.e. time-space) and, therefore, without information on the representativeness and generalization of the population values estimated in this study.”

6- e) a description of how participants were recruited, and f) descriptions of where participants were recruited and where the research took place.

RESPONSE: this point was addressed in the materials and methods section (procedure). However, we include the specification that the participation did not involve any kind of payment.

Line 176-179, “Participants were contacted by surveyors in the recreation areas of the higher education institution (e.g. reading areas, indoor courtyards, library, etc.), explaining the objectives of the study and inviting them to freely respond on the spot, without payment of any kind.”.

We hope that our answers will be adequate, however, we will gladly respond to any new requirement and with the level of detail that may be necessary.

Greetings and thank you for your consideration
