# Supplementary material for: Development and evidence of validity of the HIV risk perception scale for young adults in a Hispanic-American context
Source: PLoS One. 2020 Apr 21;15(4):e0231558. doi: 10.1371/journal.pone.0231558 (PMC7173927; doi:10.1371/journal.pone.0231558)
Supplement: Attachment — Submitted filename: Response to Reviewers_2.docx [file pone.0231558.s007.docx]

Dear Editor:

First of all, I would like to thank you for the new chance given to this work, as well as the thorough and valuable guidance provided by both reviewers. The truth is that we have received the reviewers' contributions with much gratitude, since we consider that they have allowed us to improve substantially.

Given the value of the contributions received, we have tried to address each of the points noted and, in the few cases where we have not been able to do so, we have tried to explain them in detail. Additionally, duo to our idiomatic constraints, we have submitted it to a proofreading service (https://www.proof-reading-service.com/, ref. no. 202003-50329) and adopted all suggested changes, so we hope to have increased the readability, significantly.

We will now proceed to provide the answers to each of the aspects noted (Comments are highlighted in red and responses in green):

First of all, we would like to deeply thank the exhaustive review carried out by the reviewer 1 and all the contributions made, which we recognize as extremely constructive and which have resulted in a notable improvement of the initial version.

Reviewer #1: 1. The author presented a relevant theory and examples, but the way it was presented was hard to follow. Please reference other relevant work.

Response: Due to our idiomatic constraints, we have sent the work to a proofreading service (https://www.proof-reading-service.com/, ref. no. 202003-50329) and adopted all suggested changes, so we hope to have increased the readability of this paragraph significantly. Regarding the suggestion to reference other relevant works, we ask the reviewer to please specify some relevant papers or authors that we have omitted in our review.

2. The author used ESEM to explore the factor structure. After the author confirmed the factor structure with 4 items in PSu and five items in PSe, the author could have compared three different CFA models – the correlated factor model (allowing correlation between latent PSu and PSe), the second-order, and the bifactor. The author’s rationale to select ESEM over other models was not developed enough (line 252-256).

Response: Although ESEM includes elements of exploratory factor analysis, and its name usually implies that this meaning is assigned to it, it is a confirmatory strategy, as are the CFA (Asparouhov, & Muthén, 2009; Marsh, Morin, Parker, & Kaur, 2014; Marsh et al., 2009), so our two-factor ESEM model is equivalent to the CFA model, with the only particularity that it recognizes the existence of cross-loadings. However, it seems to us that the reviewer's suggestion, to incorporate the CFA model, contributes to corroborate the strength of the structure, so we have included the equivalent CFA model.

With regard to the rationality for the choice of the model , we have followed the reviewer suggestions and tried to express ourselves better in the following paragraph (lines 252-264): “According to the most common fit criteria (CFI > .95, TLI > .95, and RMSEA < .06) [50], the original model (M1) is not enough of an explanation for the observed covariations matrix (for items descriptions, loadings and cross-loadings of the 23 items version) (S3 Table), but models based on the 9 items debugged scale, with the exception of M3, show good fit standards in most of them (i.e., M2a, M2b, M4 and M5). However, both in the ESEM (M2a) and CFA (M2b) approaches the two-factor covariate model appears to be the most parsimonious model, since M4 shows a second-order factor that explains most of the perceived susceptibility (β = .70) and very few of the perceived severity (β = .18), while the general factor of M5 shows only medium loadings (λ = .29–.54) with perceived severity and mild or no perceived susceptibility (λ = .03–.32), thus not representing a general dimension that can be interpreted. For illustrative purposes, Figures 1a and 1b show the models based on the 9 items version (i.e., M2a, M2b, M3, M4 and M5) with standardized loadings.”

Asparouhov, T., & Muthén, B. (2009). Exploratory structural equation modeling. Structural equation modeling: a multidisciplinary journal, 16(3), 397-438. <https://doi.org/10.1080/10705510903008204>

Marsh, H. W., Morin, A. J., Parker, P. D., & Kaur, G. (2014). Exploratory structural equation modeling: An integration of the best features of exploratory and confirmatory factor analysis. *Annual review of clinical psychology*, *10*, 85-110. <https://doi.org/10.1146/annurev-clinpsy-032813-153700>

Marsh, H. W., Muthén, B., Asparouhov, T., Lüdtke, O., Robitzsch, A., Morin, A. J., & Trautwein, U. (2009). Exploratory structural equation modeling, integrating CFA and EFA: Application to students' evaluations of university teaching. *Structural equation modeling: A multidisciplinary journal*, *16*(3), 439-476. <https://doi.org/10.1080/10705510903008220>

3. The study assumed that there are two dimensions in the perception scale at the beginning. That is the reason why two dimensions were developed/asked with different prompts. However, the author used ESEM which assumes that each item can be explained by not only PSu, but also PSe. There is a discrepancy here. It looks like CFA fits better than exploratory study since the author already developed the item with two dimensions. Is there any reason for conducting ESEM?

Response: We understand the reviewer's concern and appreciate the comment. The reasons why we use ESEM is because, in recent years, it has become evident that most of the meassures present cross-loadings (Tóth-Király, Bõthe, Rigó, & Orosz, 2017) and the omission of these can cause trouble to identify the models (Xiao, Liu, & Hau, 2019). However, following the reviewer's suggestions, we have included the CFA model, in order to verify the stability of the proposed structure with two different techniques.

Xiao, Y., Liu, H., & Hau, K. T. (2019). A comparison of CFA, ESEM, and BSEM in test structure analysis. Structural Equation Modeling: A Multidisciplinary Journal, 26(5), 665-677.

Tóth-Király, I., Bõthe, B., Rigó, A., & Orosz, G. (2017). An illustration of the exploratory structural equation modeling (ESEM) framework on the passion scale. Frontiers in psychology, 8, 1968.

4. It is nice that the study examined different models and presented in a figure. However, the author needs to walk readers through each model and its rationale with short descriptions.

Response: We thank the reviewer for the comment, so we have provided a better explanation of the legend of each model and improved its graphic representations.

5. The author conducted the second-order model with two latent variables but I wonder how it was even possible. A second-order factor needs at least three first-order factor indicators. However, in this study, there are only two first-order factors (PSu, PSe).

Response: We understand the reviewer's concern, since the estimation of a second-order model requires at least three latent variables. However, since the number of items and the Likert options of the scales of each latent variable are comparable, we have assumed that the second order variable has an equivalent weight in each first order factor, so we have set the latent variances to 1, which allows us to identify and estimate the model. To make this situation transparent, we have noted this (“second-order CFA (M4), with first order factor variance fixed to 1”) in statistical analysis (lines 227-228) and in the legend of table 2.

6. Poor quality of figure. In addition, the author could add another model related to Table 5.

Response: The figures were improved by dividing figure one into two (1a and 1b), and representations of all the models presented in table 2 were included. Additionally, we incorporated a figure 2 to represent the model in table 5.

7. More information is required to explain the debugged version. Also, the rationale for debugged version was not presented.

Response: We have tried to clarify our explanation, along the following lines:

(lines 222-225) “Later, to make a more brief and optimized scale, a debugged version was established by, removing items on the basis of three criteria: (1) retention of strong factorial loadings (λ > .5) [51], (2) removal of redundant items [52], and (3) removal of items with strong cross-loadings (>.3) [53, 54].”

(lines 252-264) “According to the most common fit criteria (CFI > .95, TLI > .95, and RMSEA < .06) [50], the original model (M1) is not enough of an explanation for the observed covariations matrix (for items descriptions, loadings and cross-loadings of the 23 items version) (S3 Table), but models based on the 9 items debugged scale, with the exception of M3, show good fit standards in most of them (i.e., M2a, M2b, M4 and M5). However, both in the ESEM (M2a) and CFA (M2b) approaches the two-factor covariate model appears to be the most parsimonious model, since M4 shows a second-order factor that explains most of the perceived susceptibility (β = .70) and very few of the perceived severity (β = .18), while the general factor of M5 shows only medium loadings (λ = .29–.54) with perceived severity and mild or no perceived susceptibility (λ = .03–.32), thus not representing a general dimension that can be interpreted. For illustrative purposes, Figures 1a and 1b show the models based on the 9 items version (i.e., M2a, M2b, M3, M4 and M5) with standardized loadings.”

8. Overall, long sentences with lots of comma makes it hard for readers to follow along.

Response: Due to our idiomatic constraints, we have sent the work to a proofreading service (https://www.proof-reading-service.com/, ref. no. 202003-50329) and adopted all suggested changes, so we hope to have increased the readability of this paragraph significantly.

9. The author updated the discussion section, but it still needs more work. Discussion needs to include more synthesis and placing the findings in the context of the literature and how the findings contribute to the existing literature.

Response: We have given a second reading to the discussion, adding some minor changes and trying to improve the synthesis. However, if the reviewer could give us more specific guidance, we would be pleased to address it.

10. Editing is needed. (i.e., unclosed brackets, “Tabla”?)

Response: Due to our idiomatic constraints, we have sent the work to a proofreading service (https://www.proof-reading-service.com/, ref. no. 202003-50329) and adopted all suggested changes, so we hope to have increased the readability of this paragraph significantly.

Reviewer #2: Thanks for considering our recommendations and incorporating our feedback so diligently. Copy editing is recommended prior publication.

Response: We are deeply grateful to the reviewer for his generous comments and for all the support for the improvement of the manuscript. We recognize our idiomatic constraints, so we have followed the reviewer's suggestion and submitted it to a proofreading service (https://www.proof-reading-service.com/, ref. no. 202003-50329) and adopted all suggested changes, so we hope to have increased the readability, significantly.
